# Title: p38δ Regulates IL6 Expression Modulating ERK Phosphorylation in Preadipocytes

**DOI:** 10.3389/fcell.2021.708844

**Published:** 2022-01-17

**Authors:** Selene Díaz-Chamorro, Sergio Garrido-Jiménez, Juan Francisco Barrera-López, Clara María Mateos-Quirós, Guadalupe Cumplido-Laso, María Jesús Lorenzo, Ángel Carlos Román, Edgar Bernardo, Guadalupe Sabio, José María Carvajal-González, Francisco Centeno

**Affiliations:** ^1^ Universidad de Extremadura, Departamento de Bioquímica y Biología Molecular y Genética, Facultad de Ciencias, Badajoz, Spain; ^2^ Universidad de Extremadura, Departamento de Bioquímica y Biología Molecular y Genética, Facultad de Ciencias, Cáceres, Spain; ^3^ Centro Nacional de Investigaciones Cardiovasculares (CNIC), Madrid, Spain

**Keywords:** IL6, ERK, SAPK, adipocytes, Wnt5a, JNK, P38γ, p38δ

## Abstract

IL6 is an essential cytokine in metabolism regulation and for intercommunication among different organs and tissues. IL6 produced by different tissues has different functions and therefore it is very important to understand the mechanism of its expression in adipose tissue. In this work we demonstrated that IL6 expression in mouse preadipocytes, like in human, is partially dependent on Wnt5a and JNK. Using mouse preadipocytes lacking each one of the p38 SAPK family members, we have shown that IL6 expression is also p38γ and p38δ dependent. In fact, the lack of some of these two kinases increases IL6 expression without altering that of Wnt5a. Moreover, we show that the absence of p38δ promotes greater ERK1/2 phosphorylation in a MEK1/2 independent manner, and that this increased ERK1/2 phosphorylation state is contributing to the higher IL6 expression in p38δ^−/-^ preadipocytes. These results suggest a new crosstalk between two MAPK signaling pathway, p38δ and ERK1/2, where p38δ modulates the phosphorylation state of ERK1/2.

## Introduction

Adipose tissue is composed of adipocytes surrounded by fibroblasts, preadipocytes, endothelial cells, nerves, and immune cells (Cinti, 2005). Initially it was thought that adipose tissue was merely an energy storage organ (Galic et al., 2010), however many studies in the last years have revealed its endocrine function (Kershaw and Flier, 2004; Blüher, 2013; Klöting and Blüher, 2014; Fasshauer and Blüher, 2015). All the cell types of adipose tissue are susceptible to secreting factors (metabolites, lipids, peptides) that have been generically referred to as adipokines (Ouchi et al., 2003, 2011; Berg and Scherer, 2005). These exert their actions in different organs (Blüher and Mantzoros, 2015) and regulate a great variety of biological processes like immune response, inflammation, glucose and lipid metabolism, adipogenesis, among others (Fasshauer and Blüher, 2015). Indeed, dysfunction of the adipose tissue has a central role in associated pathologies with metabolic diseases such as obesity, type 2 diabetes, cardiovascular disorders, and different cancers (Van Gaal et al., 2006; LeRoith et al., 2008; Blüher, 2013; Fasshauer and Blüher, 2015; Manieri et al., 2019).

Among 600 potential adipokines (Kershaw and Flier, 2004), IL6 has deserved a special interest because it could be related to insulin resistance and hence associated with obesity. Thus, high levels of plasma IL6 are correlated with obesity (Fried et al., 1998), type 2 diabetes and even with the prediction of this pathology (Pradhan, 2001), and it decreases with the loss of adipose tissue and body weight (Ziccardi et al., 2002; Esposito et al., 2003).

Wnt secreted glycoproteins family are key mediators of both, adipogenesis and IL6 expression. Canonical Wnt ligands (β-catenin dependent pathway) like Wnt10b have been shown to inhibit adipogenesis (Ross, 2000; Kawai et al., 2007; Takada et al., 2009), and Wnt5b (a non-canonical Wnt ligand) indirectly promotes adipogenesis by inhibiting the canonical Wnt10b pathway (Kanazawa et al., 2005). The non-canonical Wnt5a inhibits the ability of PPARγ to transcriptionally activate its downstream metabolic target genes in mesenchymal stem cells promoting osteogenesis instead of adipogenesis (Kennell and MacDougald, 2005; Cristancho and Lazar, 2011).

Conventional MAP Kinases are serine, threonine and tyrosine kinases evolutionarily conserved in all eukaryotes and play a key role in the regulation of diverse cellular programs such as proliferation, differentiation, and death, as well as in the regulation of stress responses (Cargnello and Roux, 2011; Yang and Huang, 2015)). MAPK cascades are triple kinase pathways that include a MKKK (MAPK kinase kinase), a MKK (MAPK kinase) and a terminal MAPK (Raman et al., 2007). p38 and JNK are the MAPK mainly activated by stress (Kuma et al., 2005; Manieri and Sabio, 2015). JNK is activated by MKK4 and MKK7, whereas the p38 pathway is triggered primarily by MKK3 and MKK6 (Sacks et al., 2018). The JNK family has three isoforms, JNK1, 2 and 3 (Sabio and Davis, 2010) and the p38MAPK family has four, p38α, β, γ and δ (Sacks et al., 2018). ERK is the MAPK mainly activated in response to growth factors. The ERK family has three isoforms, ERK1 and ERK2 activated by MEK1/2, and ERK5 activated by MEK5 (Cargnello and Roux, 2011).

It has been shown that ablation of JNK1 in adipose tissue decreases IL6 expression and protects against liver insulin resistance induced by high caloric diet in mice (Sabio et al., 2008). Otherwise, it was demonstrated that Wnt5a increases IL6 expression through JNK activation in obese human visceral adipocytes (Zuriaga et al., 2017). These results suggest that IL6 expression mediated by JNK could be a widespread and common mechanism in different organisms and tissues. ERK1/2 has also been implicated in IL6 expression. Human preadipocytes treated with D-dopachrome tautomerase, a novel adipokine, increase ERK1/2 phosphorylation and IL6 expression that can be attenuated by U0126 an ERK1/2 inhibitor (Ishimoto et al., 2012). In the adipocyte line 3T3-L1 and in mouse it was observed that ursolic acid treatment increases IL6 expression through ERK1/2 and NFkB (Feng et al., 2020). Although it is also known the role of p38 and ERK1/2 in the regulation of IL6 expression in various cell lines (Beyaert et al., 1996), as well as in chondrocytes (Rasheed et al., 2011), human endothelial cells (Liu et al., 2009), astrocytes (Van Wagoner et al., 2002), or smooth muscle fibers (Amrani et al., 2001), much is unknown about the role of p38 in regulating IL6 expression in adipocytes. It has recently been shown in the 3T3-L1 subjected to inflammatory stress with TNFα that MAPK (p38, JNK and ERK1/2) are activated in preadipocytes and adipocytes. By silencing the expression of dual-specificity phosphatases was observed an increase in the phosphorylated states of MAPK and the corresponding increase in the expression of pro-inflammatory genes, including IL6 (Ferguson et al., 2019).

In this work we have studied the role of p38s kinases on IL6 expression in mouse white preadipocytes. We have used immortalized preadipocytes wild type (Wt) or knockout of each p38 family member (p38α, p38β, p38γ and p38δ) to show that IL6 expression is Wnt5a and JNK dependent. Moreover, we demonstrated that the IL6 expression is mainly p38γ and p38δ dependent. In fact, the lack of some of these two kinases increases IL6 expression without altering that of Wnt5a. We show that the absence of p38δ promotes greater ERK phosphorylation in a MEK 1/2 independent manner, and this increased ERK phosphorylation state is a main contributor to the higher IL6 expression in p38δ^−/-^ mouse preadipocytes.

## Materials and Methods

### Cell Culture

White preadipocytes from wild type mice (Wt) and knockout (KO) mice lacking p38α (p38α^−/−^), p38β (p38β^−/−^), p38γ (p38γ^−/−^), p38δ (p38δ^−/−^), MKK3 (MKK3^−/−^) and MKK6 (MKK6^−/−^) kinases were immortalized by infection with SV40TpBABE-neo virus as previously described (Matesanz et al., 2017). The validity of these cell culture model has been previously supported (Matesanz et al., 2017, 2018). In any case, we have reconfirmed the KO of MKK3 and MKK6 by western blot and those of p38 by qPCR (Supplementary Figure S1).

Cells were grown in Dulbecco’s Modified Eagle Medium (DMEM, Gibco) supplemented with 10% fetal bovine serum (FBS, Gibco), l-glutamine (2 mM, Gibco), streptomycin (100 μg/ml, Gibco) and penicillin (100 U/ml, Gibco) and incubated at 37°C under a 5% CO_2_/95% air atmosphere. Confluent cells were trypsinized and seeded in tissue dishes at a density of 6 × 10^5^ cells/ml. After 8 h, the medium was aspirated and replaced with fresh medium without sera. After 4–5 h, the medium was replaced with fresh medium containing DMSO (control cells) or the indicated concentrations of BIRB 0796 (Cell Signaling), Box5 (EMD Millipore), JNK-IN-8 (Calbiochem), U0126 (Cell Signaling), PD184352 (Tocris), Microcystin L-R (Sigma) or recombinant Wnt5a (R&D Systems) and the incubation was continued for a further 8 h.

### Gene Expression Analysis by Quantitative RT-qPCR

Total RNA from culture cells was obtained using Trizol^TM^ Reagent (Thermo Fisher Scientific) following manufacture instructions. RNA (400 ng) was reverse transcribed to complementary DNA with High-Capacity cDNA reverse transcription Kit (Applied Biosystems). Gene expression was analyzed by qPCR using PowerUp Sybr Green probe (Applied Biosystems) and the appropriated primers in the QuantStudio3 (Applied Biosystems by Thermo Fisher Scientific). Relative mRNA expression was normalized to Gapdh, β-actin and E1F1a mRNA measured in each sample. The pairs of primers (forward and reverse, respectively) used in this work were the follows: 5′-TGC​AAG​AGA​CTT​CCA​TCC​AG-3′ and 5′-ATT​TCC​ACG​ATT​TCC​CAG​AG-3′ for IL6, 5′-CTG​GCA​GGA​CTT​TCT​CAA​GG-3′ and 5′-GTC​TCT​CGG​CTG​CCT​ATT​TG-3′ for Wnt5a, 5′-GCT​TTT​GAT​ACA​AAG​ACG​GGG​C-3′ and 5′-CAG​ACG​CAA​CTC​TCG​GTA​GG-3′ for p38α, 5′- CTC​CTT​GGA​AGA​ATG​CTG​GT-3′ and 5′- TTC​CAC​TCC​TCC​AGC​GTG-3′ for p38β, 5′- CAA​CAA​GGT​GGC​CAT​CAA​GA-3′ and 5′- CTC​GTG​GCG​CAT​GTG​TTT-3′ for p38γ, 5′- GTT​TGA​GAT​CTC​TTT​GTA​GAT​GTG​TTG-3′ and 5′- GGA​CCC​TGA​GGA​GGA​GAC​A-3′ for p38δ, 5′-TGA​AGC​AGG​CAT​CTG​AGG​G-3′ and 5′-CGA​AGG​TGG​AAG​AGT​GGG​A-3′ for Gapdh, 5′-TGT​TAC​CAA​CTG​GGA​CGA​CA-3′ and 5′-GGG​GTG​TTG​AAG​GTC​TCA​AA-3′ for β-actin and 5′-AAT​GTG​CTT​TGA​CGG​TGT​GA and 5′-TGA​TTT​TGG​CAT​GTT​CTG​GA-3′ for Eif1a. Reverse transcription was set at 25°C for 10 min, 37°C for 120 min and 85°C for 5 min qPCR reactions were performed at 50 °C for 2 min, 95°C for 10 min, and 50 cycles of 95°C for 15 s and 60°C for 1 min. Melt curves analysis were used to verify the specificity of each pair of primers. Relative gene expression was calculated with the *ΔΔCt* method. The expression of the target mRNA was normalized to the average mRNA expression of the three reference genes β-actin, Eif1a and Gapdh.

### Western Blot

Treated and control cells were lysed in ice-cold lysis buffer containing 50 mM Tris-HCl (pH 7.5), 1 mM EGTA, 1 mM EDTA, 1 mM sodium orthovanadate, 10 mM sodium fluoride, 5 mM sodium pyrophosphate, 0.27 M sucrose, 0.1 mM phenylmethylsulphonyl fluoride, 1% (v/v) Triton X-100, 0.1% (v/v) 2-mercaptoethanol and complete protease inhibitor cocktail. After lysis, cell debris was removed by centrifugation at 20000 g for 30 min at 4°C and protein concentrations in the supernatants were determined using the Bio-Rad protein assay, according to the instructions of the manufacturer. Equal amounts of protein (15 µg) were separated on 10% SDS-PAGE and blotted onto nitrocellulose membranes (Bio-Rad Laboratories, Hercules, CA, United States). The membranes were then blocked with 5% non-fat dry milk Tris-buffered saline (pH 7.5) containing 0.05% Tween-20 and incubated with appropriate primary and horseradish peroxidase (HRP)-conjugated secondary antibodies in blocking buffer [5% non-fat dry milk in Tris-buffered saline (pH 7.5) containing 0.05% Tween-20 (TBS-T)]. Antibodies used were anti-phosphoC-Jun (Cell Signaling #3270, 1:1,000), anti-C-Jun (Cell Signaling #9165, 1:1,000), anti-phosphoJNK (Cell Signaling #4668, 1:1,000), anti-JNK (Cell Signaling #9252, 1:1,000), anti-MKK3 (Cell Signaling #8535, 1:1,000), anti-MKK6 (Cell Signaling **#**9264, 1:1,000), anti-pERK1/2 (Cell Signaling #9101, 1:1,000), anti-ERK1/2 (Cell Signaling #9102, 1:1,000), anti-αtubulin (Thermo Fisher, #32–2,500, 1:1,000), anti-vinculin (Sigma, #V4505, 1:1,000), anti-rabbit-HRP (Cell Signaling #7074, 1:1,000) and anti-mouse-HRP (Cell Signaling #7076, 1:1,000). After required washes with TBS-T, proteins were analyzed using an enhanced chemiluminescence detection system (SuperSignal West Dura, Thermo Fisher) and iBright CL1000 (Invitrogen by Thermo Fisher Scientific). For quantification, images were processed using ImageJ software (Fiji) and Adobe Photoshop CC 2018. Protein levels were normalized to αtubulin or vinculin (loading controls) for each of the samples and phospho-protein levels were made relative to total-protein previously normalized to tubulin or vinculin for each of the samples.

### Protein Target Prediction

With the information of BIRB796 obtained in PubChem (https://pubchem.ncbi.nlm.nih.gov/), we accessed to SwissTargetPrediction (http://www.swisstargetprediction.ch/) (Daina et al., 2019) using *Mus musculus* as reference to find the probability of a match between BIRB796 and targets.

### Statistical Analysis

Data were analyzed using unpaired two-tailed *t*-test for two group comparisons and one-way ANOVA coupled to Bonferroni’s post-test for three or more groups comparisons. The n value in figure legends for Western-blots and qPCR represents independent samples. In qPCR experiments, each independent sample is the average of 2–3 technical replicates. Data with a *p*-value < 0.05 were considered significant. Statistical analysis was performed with GraphPad Prism Software v7.00.

## Results

### JNK Controls IL6 Expression in Wt Preadipocytes

**FIGURE 1:**
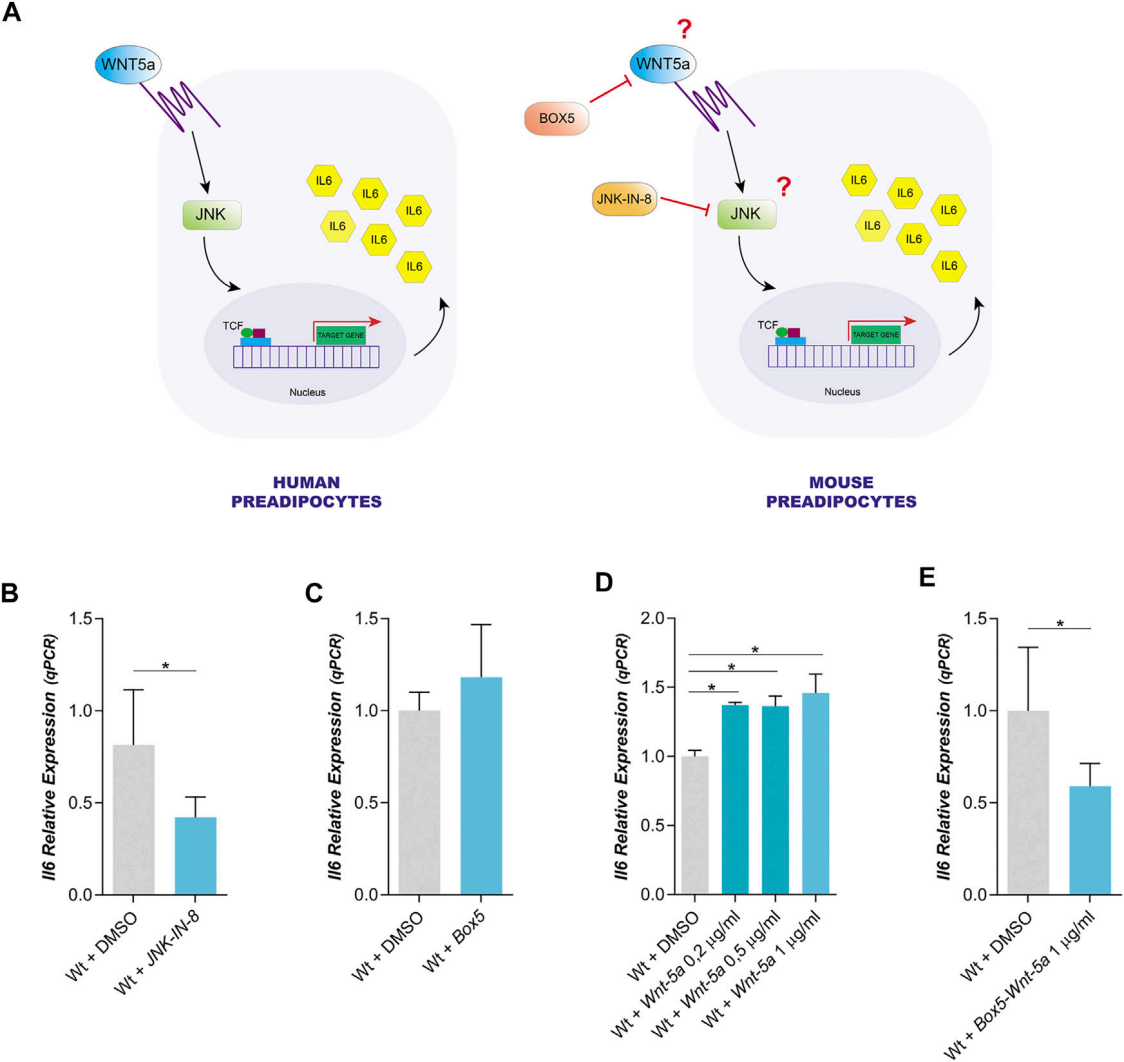
Dependence of IL6 expression on Wnt5a and JNK in mouse Wt preadipocytes. **(A)**. Model of IL6 expression proposed for human Wt preadipocytes versus the same model of IL6 expression in mouse preadipocytes. **(B)**. Effect of JNK-IN-8 (3µM) (a JNK inhibitor) treatment on IL6 expression in Wt preadipocytes (*n* = 4). **(C)**. Relative IL6 expression in mouse Wt preadipocytes treated with Box5 (3 μg/ml) (a Wnt5a antagonist) (*n* = 2), **(D)** or treated with different concentrations of recombinant Wnt5a (0,2 μg/ml; 0,5 μg/ml and 1 μg/ml) (*n* = 2) E or co-treated with Box5 (3 μg/ml) and recombinant Wnt5a (1 μg/ml) (*n* = 4). *p*-value were obtained using unpaired two-tailed *t*-test or one-way ANOVA coupled to Bonferroni’s post-test. *p < 0.05.

In human preadipocytes, the expression of IL6 is dependent on Wnt5a through the activation of JNK signaling pathway (Figure 1A) (Zuriaga et al., 2017). It is not yet known whether the expression of IL6 mediated by Wnt5a/JNK is an extended mechanism to adipocytes of other species, including the mouse which is a model in many studies on the role of adipocytes in obesity and the associated pathologies to this disease (Figure 1A).

We have used immortalized mouse preadipocytes to study the expression of IL6 and Wnt5a by qPCR. In Wt preadipocytes, IL6 expression was reduced by the inhibition of the JNK pathway with the cell-permeable JNK peptide inhibitor (JNK-IN-8), but it was not altered by an antagonist of Wnt5, Box5 (Figures 1B,C). These results could suggest that endogenous Wnt5a expression level is insufficient to induce IL6 expression. Furthermore, Wt preadipocytes expressed Wnt5 and this expression was not affected by the Wnt5a antagonist or by JNK inhibition (Supplementary Figure 2A). The effect of the inhibitor on JNK activity was assessed by analyzing the phosphorylation state of c-Jun, a canonical substrate of JNK. We observed that JNK-IN-8 was efficient in blocking the phosphorylation of c-Jun (Supplementary Figure 2B). In fact, the exogenous addition of recombinant Wnt5a did not alter its own expression (Supplementary Figure 2A), but it produced an increase in IL6 expression in Wt preadipocytes (Figure 1D). The expression of IL6 was not affected by Box5 (Figure 1C) but it was decreased by Box5 and Wnt5a (Figure 1E), suggesting that IL6 expression could be dependent on Wnt5a.

### p38 SAPKs Regulate IL6 Expression in Mouse Preadipocytes

In addition to JNK pathway, p38s SAPKs could also regulate IL6 expression (Beyaert et al., 1996; Amrani et al., 2001; Van Wagoner et al., 2002; Liu et al., 2009; Rasheed et al., 2011; Zur et al., 2015; Rajamäki et al., 2016; Cuenda and Sanz-Ezquerro, 2017). Therefore, we decided to study whether these SAPK may be involved in the expression of IL6 and Wnt5a in mouse preadipocytes.

**FIGURE 2:**
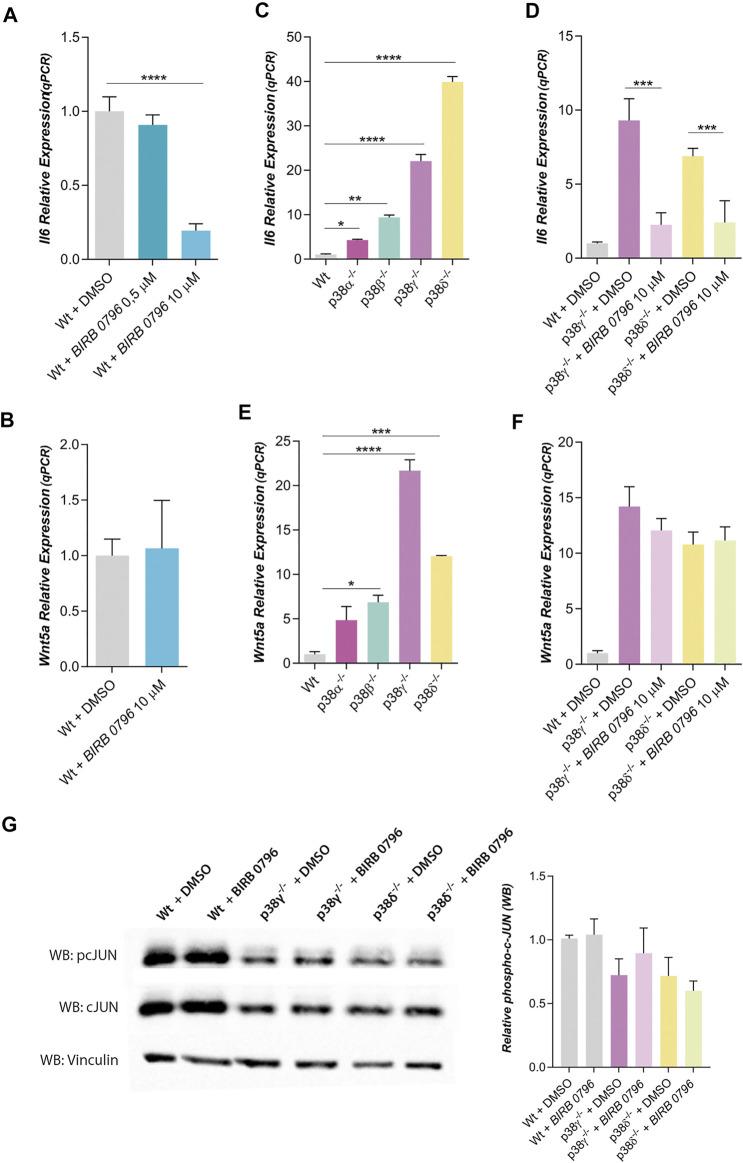
Dependence of IL6 and Wnt5a expression with p38 family kinases. **(A,B)** corresponds to relative IL6 and Wnt5a expressions in mouse Wt preadipocytes treated with BIRB 0796 (an inhibitor of the p38 kinases) (*n* = 2). **(C,D)** correspond to relative IL6 expression in Wt and p38s KO mouse preadipocytes (*n* = 2) and their dependence with BIRB 0796 treatment (n = 4). **(E)** and F correspond to Wnt5a expression in Wt and p38s KO mouse preadipocytes and their dependence with BIRB 0796 treatment (*n* = 2). **(G)**. Western blots and quantification of relative phosphorylation state of cJUN (pcJUN) in control adipocytes and treated with BIRB 0796 (*n* = 4). Total cJUN and vinculin (loading control) were used to the quantification of pcJUN. Unless otherwise stated, the concentration of BIRB 0796 used in the pretreatments were 10 μM. Bars represent the mean of gene expression in each condition and error bars represent the standard deviation. *p*-value were obtained using unpaired two-tailed *t*-test or one-way ANOVA coupled to Bonferroni’s post-test. *p < 0.05, **p < 0.01, ***p < 0.001, ****p < 0.0001.

In wild type mouse preadipocytes, IL6 expression was strongly reduced when all four p38 family members were inhibited with BIRB 0796 (10 μM), but it was not altered when only p38α and β were inhibited with BIRB 0796 (0.5 μM) (Kuma et al., 2005) (Figure 2A). However, the inhibition of the four p38 family members did not change Wnt5a expression (Figure 2B). These results suggest that p38γ and δ would be regulating IL6 expression but not Wnt5a expression in mouse preadipocytes.

To discern the role of p38 family members in IL6 expression, we have used KO mouse preadipocytes for each one of them. Thus, we observed that IL6 expression was increased in all of them, notably in preadipocytes lacking p38γ or δ (Figure 2C). The inhibition of all p38s (with BIRB 0796) blocked the increase of IL6 expression in preadipocytes p38γ^−/−^ or δ^−/−^ (Figure 2D). Wnt5a expression was also increased in KO preadipocytes (Figure 2E), but this expression of Wnt5 was not altered by BIRB 0796 treatment (Figure 2F). Since it has been shown that BIRB 0796 at 10 µM can also inhibit JNKs and not only p38s (Bain et al., 2007; Kuglstatter et al., 2010), we have studied if JNKs were being inhibited by BIRB 0796 in KO adipocytes. As shown in Figure 2G, c-Jun phosphorylation was not affected by BIRB 0796. Therefore, the increase of IL6 expression in preadipocytes p38γ^−/-^ or δ^−/-^ was not mediated by JNK inhibition by BIRB 0796. Then, our results suggested that the lack of p38γ or δ increases Wnt5a and IL6 expression in mouse preadipocytes.

**FIGURE 3:**
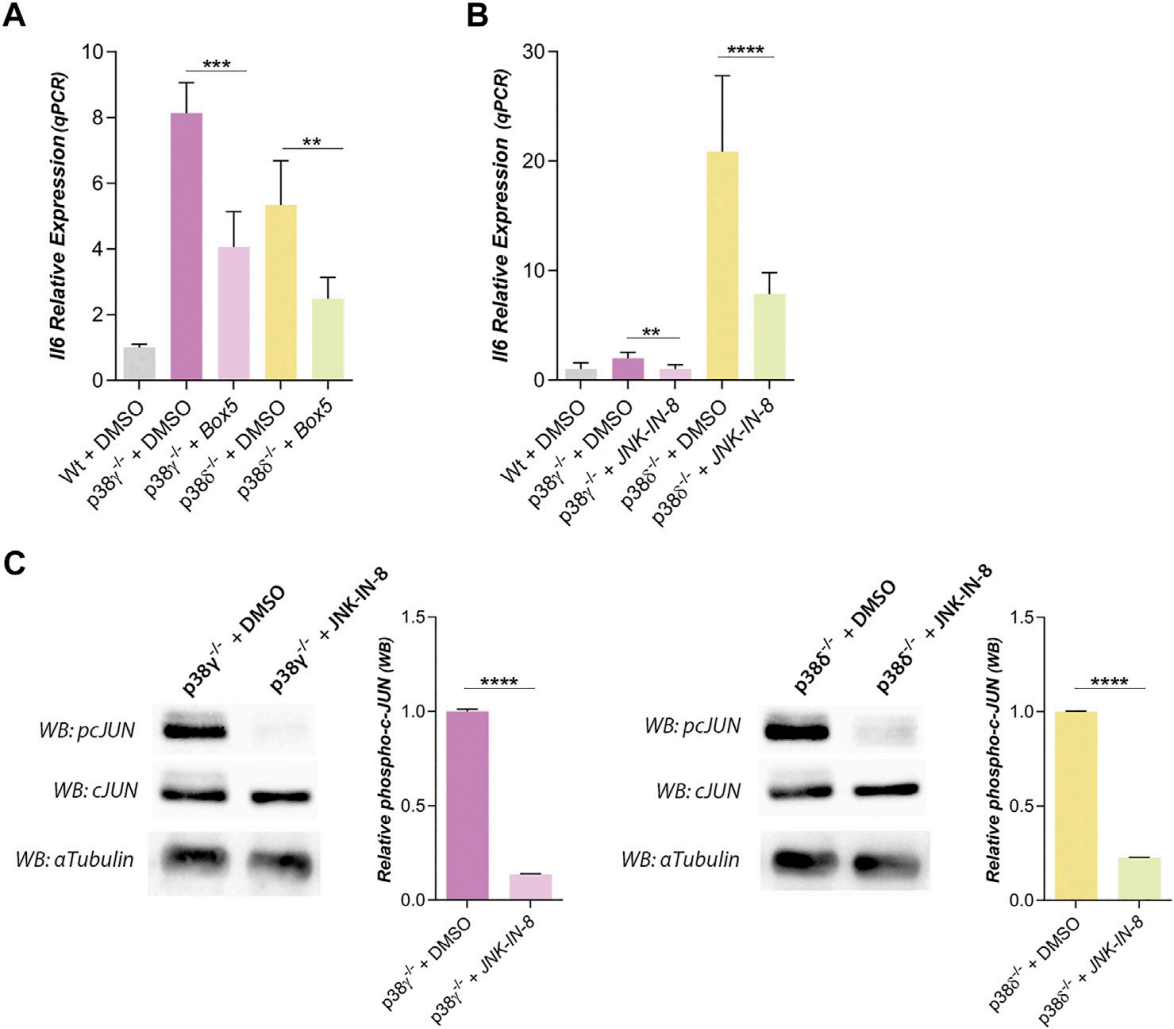
IL6 expression is regulated by Wnt5a and JNK signaling pathway in preadipocytes p38γ^−/−^ and δ^−/−^. **(A)**. Relative IL6 expression of preadipocytes treated with Box5 (3 μg/ml), an antagonist of Wnt5a (*n* = 3), and **(B)** treated with JNK-IN-8 (3 µM), a JNK inhibitor (*n* = 6). **(C)**. Western blots and quantification of phospho-cJUN (pcJUN), in control adipocytes and treated with JNK-IN-8 (3 µM) (*n* = 2). Total cJUN and α-tubulin (loading control) were used to the quantification of pcJUN. *p*-value were obtained using one-way unpaired two-tailed *t*-test or one-way ANOVA coupled to Bonferroni’s post-test. **p < 0.01, ***p < 0.001, ****p < 0.0001.

To determine whether the increase of Wnt5a expression was responsible of IL6 induction, cells were treated with Box5, an antagonist of Wnt5a (Figure 3A). Thus, Box5 significantly reduced IL6 expression in p38γ^−/−^ or p38δ^−/−^ preadipocytes. These results suggest that expression of IL6 was regulated by Wnt5a. It was described that IL6 expression was Wnt5a dependent and modulated by JNK. Then, we studied the IL6 expression of preadipocytes treated with a soluble peptide inhibitor of JNK (Figure 3B). In this condition, the expression of IL6 was reduced with respect to its controls in preadipocytes p38γ^−/−^ or δ^−/−^ (Figure 3B). The effect of the JNK inhibitor was assessed by analyzing the phosphorylation state of c-Jun. As shown in Figure 3C; Supplementary Figure S3, JNK-IN-8 was efficient in blocking the phosphorylation of c-Jun. Moreover, the phosphorylation state of JNK in WT and KO preadipocytes for p38γ or δ was the same (Supplementary Figure S4). Therefore, these results suggested that the lack of p38γ or δ increases the Wnt5a expression in mouse preadipocytes, which in turn might promote greater IL6 expression. This process would be mediated by p38 signaling pathway and JNK is necessary for the transcription of IL6.

### p38δ Could Regulate the Phosphorylation State of ERK 1/2

**FIGURE 4:**
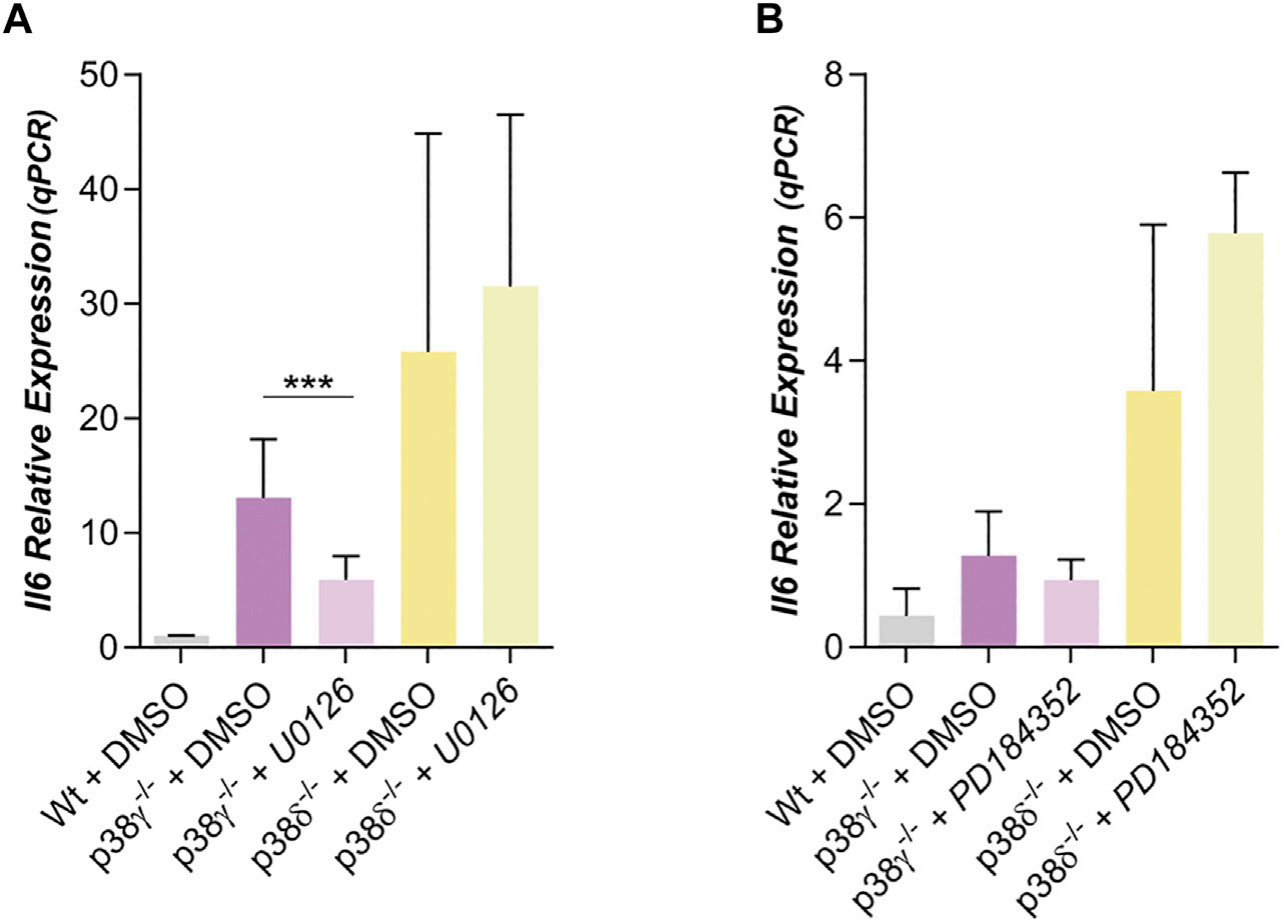
Dependence of IL6 with ERK in preadipocytes p38γ^−/−^ or δ^−/−^. **(A)**. Relative IL6 expression in preadipocytes p38γ^−/-^ or δ^−/-^ treated with U0126 (10 µM) (*n* = 5) **(B)** or with PD184352 (2 µM) (*n* = 4) (both MEK 1/2 inhibitors). *p*-value were obtained using one-way ANOVA coupled to Bonferroni’s post-test. ***p < 0.001.

In other cell types, the crosstalk between signaling pathways, notably the SAPK (p38s) with ERKs, had been suggested (Hotokezaka et al., 2002; Leaner et al., 2003; Risco et al., 2012; Zakrzewska et al., 2019). Then, we decided to study whether the ERK 1/2 signaling might be regulating IL6 and Wnt5a expression in preadipocytes. For this we have used U0126 and PD184352, selective inhibitors of MEK1/2 (Duncia et al., 1998; Mody et al., 2001; Hotokezaka et al., 2002), the MAPKKs that phosphorylates and activates ERK 1/2. IL6 expression was diminished by treatment with U0126 in the case of p38γ^−/−^ mouse preadipocytes (Figure 4A) and in wild type preadipocytes (Supplementary Figure S5A), while IL6 expression in p38δ^−/−^ preadipocytes was not altered by U0126 (Figure 4A). Through western blot it was observed that treatment with this MEK 1/2 inhibitor decreased phospho- ERK 1/2 in white wild type and p38γ^−/−^ preadipocytes, but not in p38δ^−/−^ preadipocytes (Supplementary Figure 5B). However, when PD184352 was used to inhibit MEK 1/2 and then ERK 1/2, we observed that it did not significantly alter the expression of IL6 neither in Wt (Supplementary Figure S5A) nor in p38γ^−/−^ nor p38δ^−/−^ preadipocytes (Figure 4B), despite blocking ERK phosphorylation (Supplementary Figure S5C) and reducing expression by Wnt5a (Supplementary Figure S5D). The fact that the results obtained with PD184352 and U0126 did not coincide, and mainly that U0126 inhibited IL6 expression, together with the fact that the phosphorylation state of ERK1/2 was the same in all adipocytes (Supplementary Figure S6) suggests that this effect could be mediated by another protein than ERK1/2 but would be inhibited by U0126, and that its inhibition would allow that ERK1/2 remained phosphorylated in p38δ^−/−^ adipocytes.

### p38δ Could Regulates IL6 Expression Regulating ERK Phosphorylation State

MKK3 and MKK6 are specific p38s activators. Since MKK3 phosphorylates and activates mainly p38δ, although p38β and γ are also their targets, and MKK6 phosphorylates and activates indistinctly and preferably p38β and γ (Manieri and Sabio, 2015; Cuenda and Sanz-Ezquerro, 2017), we studied Wnt5a and IL6 expression in mouse white preadipocytes MKK3^−/−^ or MKK6^−/−^.

**FIGURE 5:**
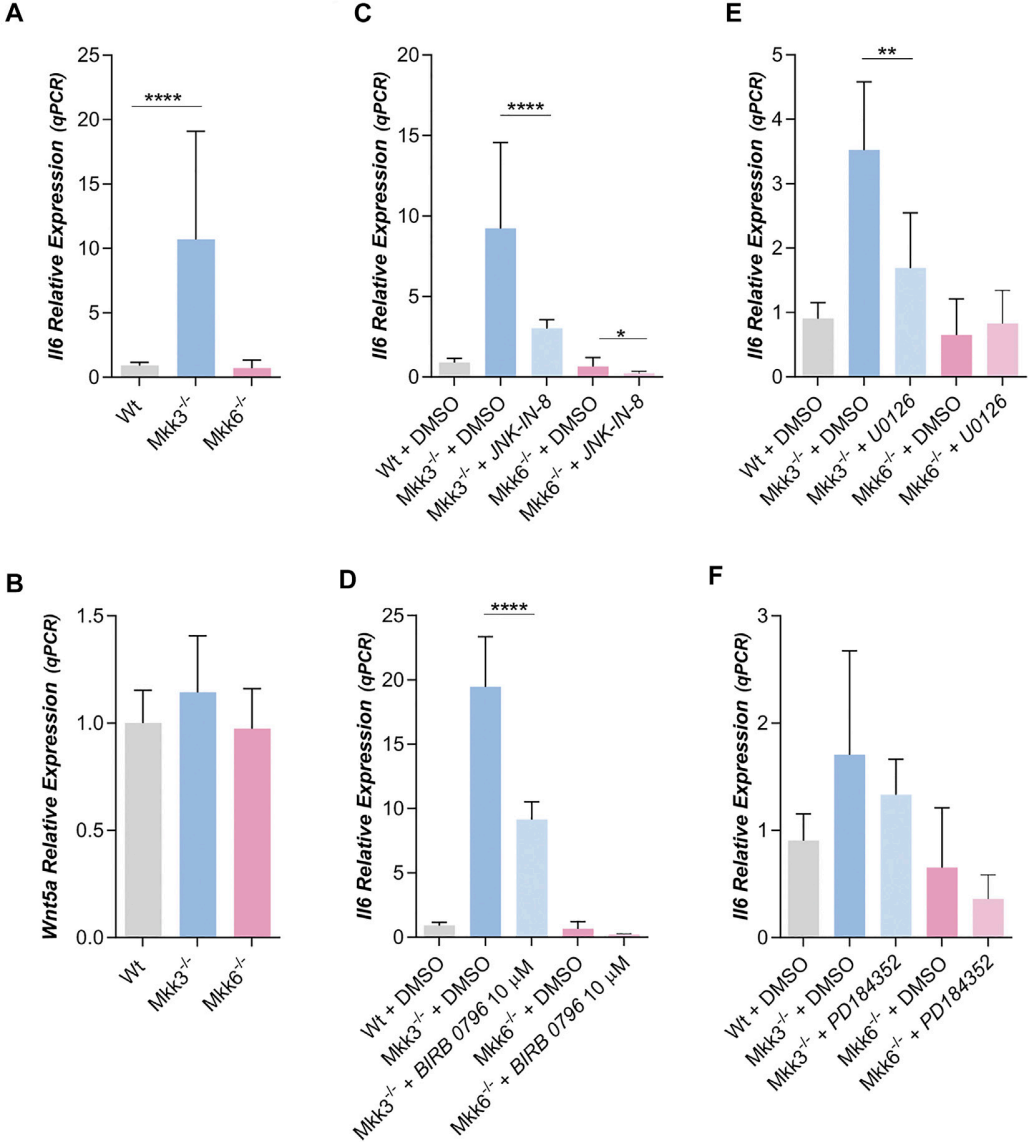
IL6 and WNT5a expression in MKK3^−/−^ and MKK6^−/−^ mouse preadipocytes. **(A)** Relative IL6 (n = 30) and **(B)** Wnt5a (*n* = 2) expression in Wt, MKK3^−/−^ and MKK6^−/−^ mouse preadipocytes. **(C)**. Relative IL6 expression in preadipocytes treated with JNK-IN-8 (3 µM) (*n* = 4) or **(D)** treated with BIRB 0796 (10 µM) (*n* = 2). **(E)**. Effect of the treatment with U0126 (10 µM) (*n* = 4) **(F)** or with PD184352 (2 µM) (*n* = 4) (both MEK 1/2 inhibitors) on IL6 expression in MKK3^−/−^ and MKK6^−/−^ preadipocytes. *p*-value were obtained using one-way ANOVA coupled to Bonferroni’s post-test. *p < 0.05, **p < 0.01, ****p < 0.0001.

IL6 expression was strongly increased in preadipocytes lacking MKK3 versus IL6 expression in Wt. The level of IL6 expression in preadipocytes lacking MKK6 is like Wt (Figure 5A). Moreover, Wnt5a expression was very similar in preadipocytes Wt, MKK3^−/−^ and MKK6^−/−^ (Figure 5B). Moreover, IL6 expression in preadipocytes MKK3^−/−^ and MKK6^−/−^ was not altered by treatment with Box5 or exogenous added Wnt5a (Supplementary Figures S7A, S7B). Thus, the increase of IL6 expression in MKK3^−/−^ preadipocytes probably was independent of Wnt5a expression. Moreover, IL6 expression in preadipocytes MKK3^−/−^ and MKK6^−/−^ was affected by treatment with JNK-IN-8 (Figure 5C) decreasing JNK activity (Supplementary Figure S6E), suggesting again that JNK is necessary for IL6 expression.

Treatment with BIRB 0796 significantly decreased IL6 expression in mouse preadipocytes MKK3^−/−^, but not in mouse preadipocytes MKK6^−/−^ (Figure 5D). Since MKK3 mainly phosphorylates p38δ, these results against suggest a role of p38δ in IL6 expression mediated by ERK 1/2, although other p38s, like β and γ could also participate.

U0126 treatment of mouse MKK3^−/−^ preadipocytes also decreases IL6 expression but not in MKK6^−/−^ (Figure 5E). In this regard, we observed that U0126 decreased phospho-ERK in both mouse preadipocytes, MKK3^−/−^ and MKK6^−/−^ (Supplementary Figure 7C). However, PD184352 treatment of mouse MKK3^−/−^ and MKK6^−/−^ preadipocytes did not decreases IL6 expression (Figure 5F), but PD184352 decreased phospho-ERK 1/2 in both mouse preadipocytes, MKK3^−/−^ and MKK6^−/−^ (Supplementary Figure 7D). That is, at least in MKK3^−/−^ preadipocytes, IL6 expression again depends on the activity of a protein different of ERK 1/2 but inhibited by U0126.

**FIGURE 6:**
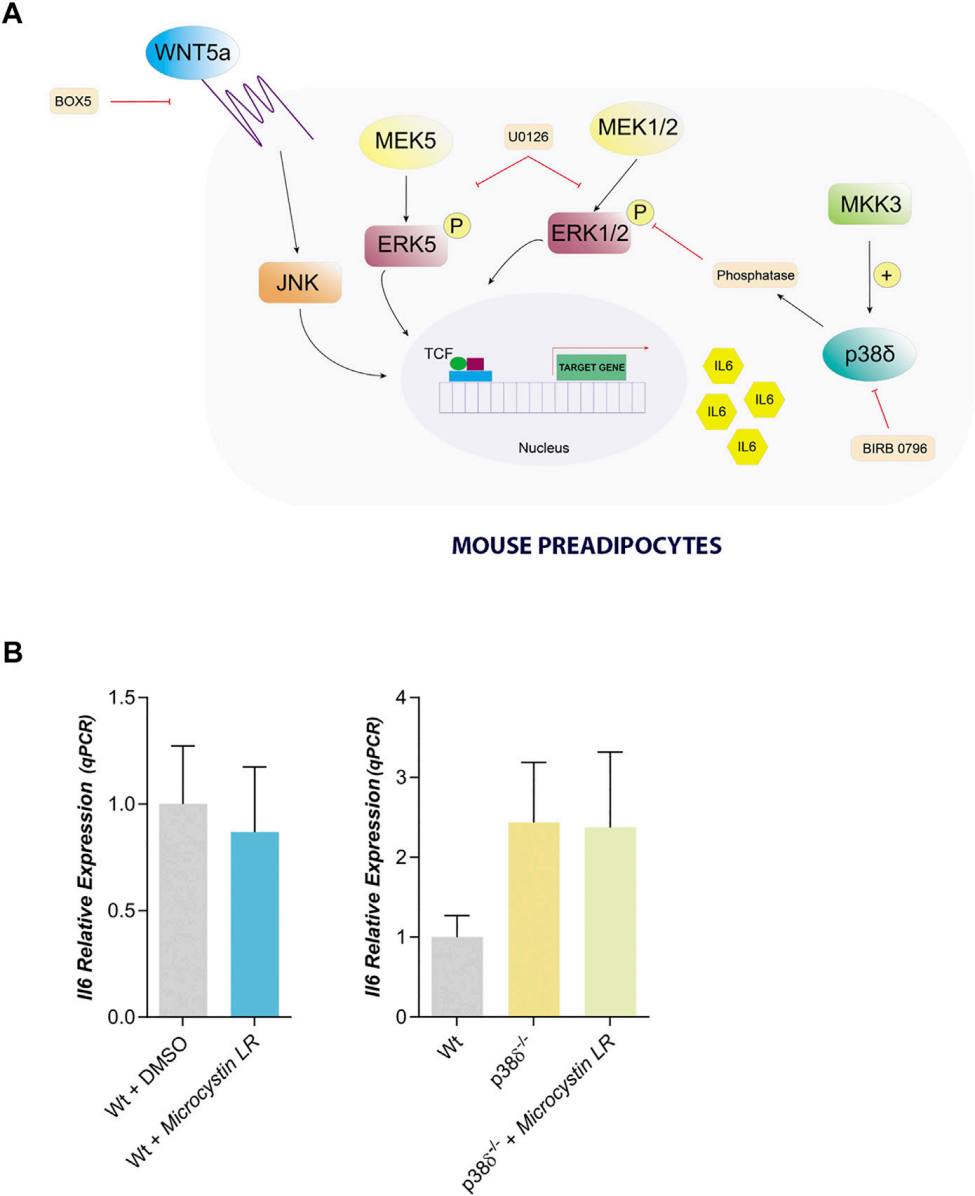
Proposed model to explain IL6 expression in mouse preadipocytes. **(A)**. IL6 expression can be mediated by Wnt5a through JNK and can be blocked by Box5. Moreover, in mouse preadipocytes we founded a crosstalk between ERK1/2 and p38δ regulating IL6 expression, in such way that the lack of p38δ favors that ERK1/2 remains active. Therefore, we propose that p38δ must be activating an ERK protein phosphatase. This signalling pathway can be blocked by U0126, that also can be inhibiting ERK5. Our results suggest that ERK5 might also be regulating IL6 expression. **(B)**. IL6 relative expression in Wt and p38δ^−/−^ preadipocytes treated with Microcystin LR (10 µM) (*n* = 4) (a PP1 and PP2A phosphatase inhibitor). *p*-value were obtained using unpaired two-tailed *t*-test or one-way ANOVA coupled to Bonferroni’s post-test.

Our results showed that IL6 expression in mouse preadipocytes is regulated by JNK and by p38δ. So, in preadipocytes lacking p38δ the level of phosphorylated ERK 1/2 were higher than in control and IL6 expression would be independent of its activation by MEK 1/2. One possibility to explain these results would be that p38δ could activate a protein phosphatase which would be the one that dephosphorylate ERK 1/2 (Figure 6A). To further this point, we have treated Wt and p38 δ^−/−^ preadipocytes with microcystin, an inhibitor of protein phosphatases 1 and 2A, and IL6 expression was unaffected by this treatment (Figure 6B). These results suggest that, at least, PP1 and PP2A are not the protein phosphatases regulated by p38 δ in white mouse preadipocytes.

## Discussion

IL6 is a potent cytokine that is expressed by different tissues under very different conditions, for example, exercise causes increased IL6 expression by muscle (Fischer, 2006), insulin signaling by hypothalamic neurons causes increased hepatic expression of IL6 (Könner et al., 2007), or obesity increases IL6 expression by adipose tissue (Mohamed-Ali et al., 1997).

In this work we show that Wnt5a modulate IL6 expression in mouse preadipocytes. It has been reported before that Wnt5a is expressed in higher level in visceral adipocytes and specially in obesity, and that this increased expression correlates with higher IL6 expression in human samples (Fuster et al., 2015; Zuriaga et al., 2017). Wnt5a can activate a great variety of signaling pathways, but JNK is the main signaling kinase activated by the Wnt pathway in mammals (Boutros et al., 1998; Gros et al., 2010). Notably, experimental studies suggest that JNK signaling is particularly relevant in the setting of obesity-induced inflammation and associated metabolic dysfunction (Sabio et al., 2008; Han et al., 2013). Our results also show that JNK signaling pathways is implicated in IL6 expression mediated by Wnt5a in mouse preadipocytes.

The p38 family kinases modulate the IL6 expression, mainly the p38γ or the p38δ. It has been extensively reported in the literature that the stimulation of very diverse cell types by stresses, especially inflammatory, leads to an activation of p38s, which would mediate an increase in IL6 expression (Beyaert et al., 1996; Duncia et al., 1998; Amrani et al., 2001; Van Wagoner et al., 2002; Liu et al., 2009; Rasheed et al., 2011). Our results show that p38s must repress the expression of IL6, since its ablation induces the expression of IL6, and as we have said before, fundamentally p38γ or p38δ. Moreover, p38s kinases also negatively regulate Wnt5a expression because individuals p38 knock outs show increased Wnt5a expression which correlate with increased IL6 expression. These results are in agree with previous work (Zuriaga et al., 2017).

In this work we show that the inhibition of p38 with BIRB796 10 µM decreases the expression of IL6, a result contrary to that obtained with the KOs of p38 γ and δ. BIRB 10 µM has been shown to inhibit human p38 γ and δ, as well as to inhibit JNK (Kuma et al., 2005). Since our results show that BIRB796 do not change the phosphorylation state of JNK (Figure 2G), this last possibility is ruled out. To check if BIRB796 could inhibit mouse p38γ and δ, we used Expasy’s Protein Target Prediction bioinformatics tool and we observed that in mice the prediction is that BIRB796 would be a ligand of p38 α, β, JNK2 and ERK 1/2 (Supplementary Table S1). Therefore, it would be probable that in mouse preadipocytes BIRB796 was not inhibiting p38γ and δ, and that the inhibition of IL6 expression was due to the inhibition of another kinase that could be of the family of the ERK.

Our results also show that ERK 1/2 and IL6 are related in mouse preadipocytes because MEK inhibition with U0126 partially blocks IL6 expression in p38γ^−/-^ adipocytes, and correlate well with recent results where it was shown that ERK 1/2 phosphorylation increases IL6 production in adipocytes (Lee et al., 2019; Feng et al., 2020). Similar role of ERK 1/2 in IL6 expression was obtained in other cellular system (Budai et al., 2013; Huang et al., 2018; Hu et al., 2020).

However, whilst ERK 1/2 phosphorylation is MEK dependent in p38γ^−/−^ adipocytes, in p38δ^−/−^ adipocytes ERK 1/2 phosphorylation was not inhibited by U0126 (Figure 4A), suggesting that ERK 1/2 phosphorylation was p38δ dependent. This should be a new crosstalk between ERK 1/2 and p38 which would be mediated by a ERK phosphatase. A similar interaction has been shown recently (Hsiao et al., 2020) suggesting that curcumin activates p38 and PP2A inducing ERK dephosphorylation. Using microcystin as an inhibitor, we ruled out that the phosphatases involved could be PP1 and PP2A. Although it is a negative result, we must consider that other phosphatases could be being regulated by the p38δ in preadipocytes, and that they were responsible for the level of phosphorylation of ERK 1/2.

Finally, our results show that PD184352, another MEK 1/2 inhibitor, does not alter the level of IL6 expression in MKK3^−/−^ and MKK6^−/−^ preadipocytes, unlike we observed using U0126. PD184352 at 2 µM has been shown to inhibit phosphorylation of ERK 1/2 and not ERK5, while U0126 at 10 µM can inhibit phosphorylation of all three (Mody et al., 2001). Therefore, the inhibition of IL6 expression by U0126 in MKK3^−/−^ preadipocytes suggests that ERK5 also regulates IL6 expression. Recently it was show that ERK5 is requires for IL6 production in tumor cells. In fact, inhibition of ERK5 or its depletion prevents IL6 production (Riegel et al., 2021).

## Data Availability

The original contributions presented in the study are included in the article/Supplementary Material, further inquiries can be directed to the corresponding authors.
