# Supplementary material for: Title: p38δ Regulates IL6 Expression Modulating ERK Phosphorylation in Preadipocytes
Source: Front Cell Dev Biol. 2022 Jan 17;9:708844. doi: 10.3389/fcell.2021.708844 (PMC8802314; doi:10.3389/fcell.2021.708844)
Supplement: Supplementary file 1 [file DataSheet1.pdf]

Supplementary Figure 1

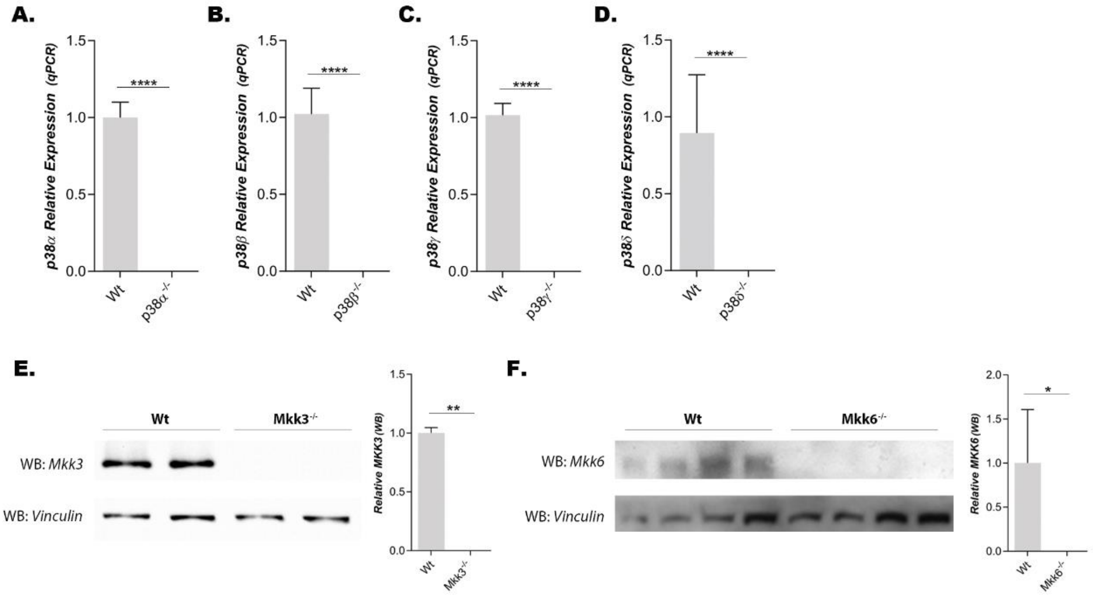

Supplementary Figure 1. **White preadipocytes from wild type mice (Wt) and knockout (KO) mice lacking p38α (p38α$^{-/-}$), p38β (p38β$^{-/-}$), p38γ (p38γ$^{-/-}$), p38δ (p38δ$^{-/-}$), MKK3 (MKK3$^{-/-}$) and MKK6 (MKK6$^{-/-}$) kinases.** A. p38α relative expression in Wt and p38α$^{-/-}$ preadipocytes (n=4). B. Relative p38β expression in Wt and p38β$^{-/-}$ preadipocytes (n=4). C. p38γ relative expression in Wt and p38γ$^{-/-}$ preadipocytes (n=4). D. Relative p38δ expression in Wt and p38δ$^{-/-}$ preadipocytes (n=4). E. Western blot and quantification of MKK3 in Wt and MKK3$^{-/-}$ preadipocytes (n=4). Total MKK3 and Vinculin (loading control) were used to the quantification of MKK3. F. Western blot and quantification of MKK6 in Wt and MKK6$^{-/-}$ preadipocytes (n=4). Total MKK6 and Vinculin (loading control) were used to the quantification of MKK6. P-value were obtained using unpaired two-tailed t-test. *$*p < 0.05$, $**p < 0.01$, $****p < 0.0001$.*

Supplementary Figure 2

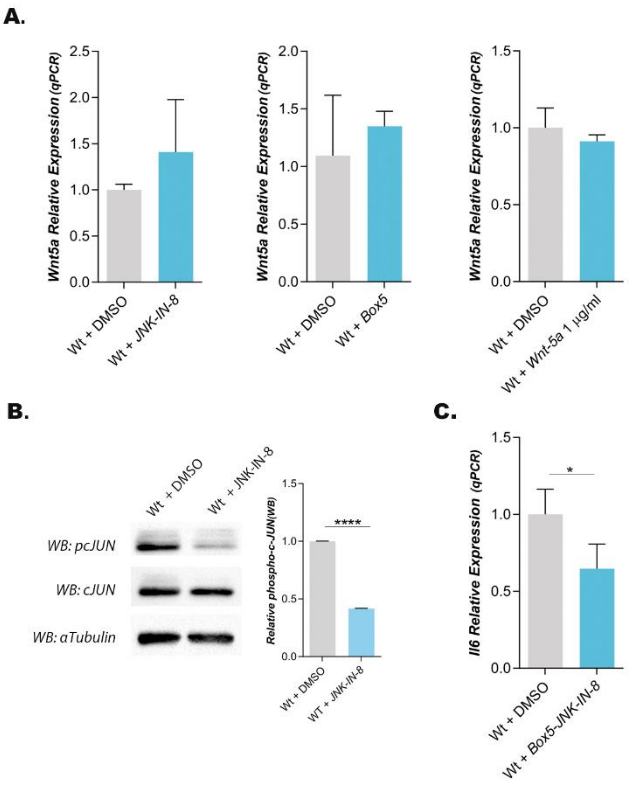

Supplementary Figure 2. Il6 and Wnt5a expression and phospho-JNK state in WT preadipocytes. (A) Relative WNT5a expression in Wt preadipocytes treated with JNK-IN-8 (3µM), a JNK inhibitor, or treated with Box5 (3µg/ml), an antagonist of Wnt5a, or treated with recombinant and exogenous Wnt5a (1µg/ml) (n=2). (B) Western blot and quantification of phospho-cJUN (pcJUN) in control Wt preadipocytes or treated with JNK-IN-8 (3 µM) (n=2). Total cJUN and α-tubulin (loading control) were used to the quantification of pcJUN. (C) Relative IL6 expression in Wt preadipocytes co-treated with Box5 (3µg/ml) and JNK-IN-8 (3 µM) (n=4). P-value were obtained using unpaired two-tailed t-test. *$*p < 0.05$, $****p < 0.0001$*.

Supplementary Figure 3

A.

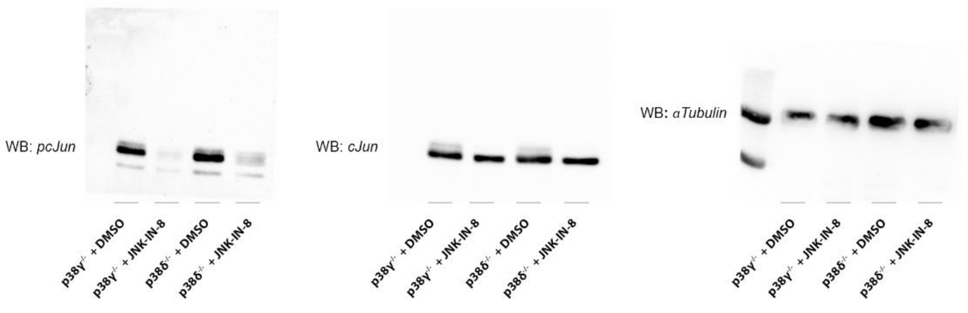

Supplementary Figure 3. Uncropped original membranes of Western blot of pcJUN in p38γ$^{-/-}$ or p38δ$^{-/-}$ preadipocytes. A. Western blot of phospho-cJUN (pcJUN), cJUN and αTubulin in control preadipocytes or treated with JNK-IN-8 (3 µM).

Supplementary figure 4

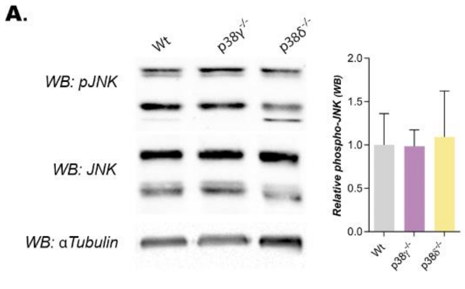

Supplementary Figure 4. Phosphorylation state of JNK in WT and p38γ$^{-/-}$ or p38δ$^{-/-}$ preadipocytes. A. Western blot and quantification of phospho-JNK (n=3). Total JNK and α-Tubulin (loading control) were used to the quantification of pJNK. P-value were obtained using one-way ANOVA coupled to Bonferroni's post-test.

Supplementary figure 5

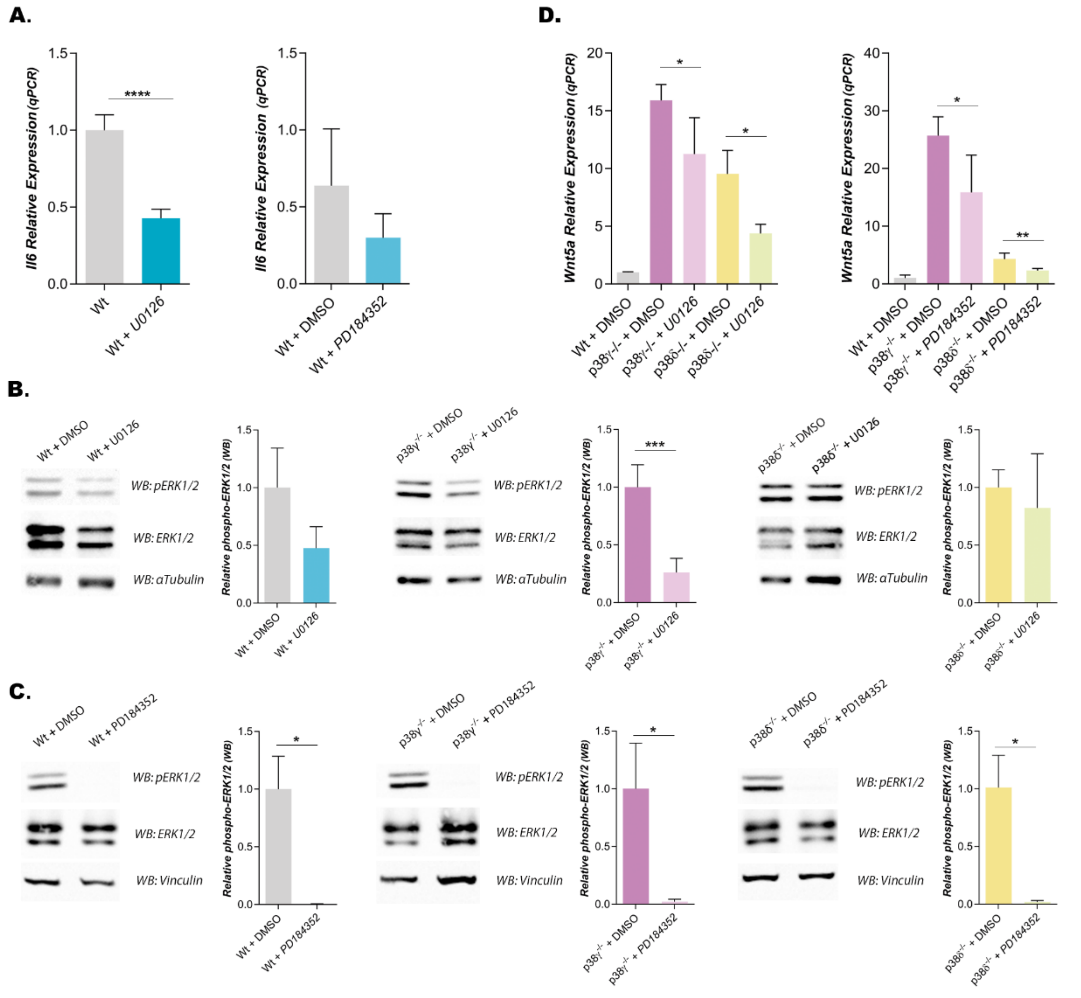

Supplementary Figure 5. IL6 and Wnt5 expression and phosphorylation state of ERK1/2 in WT, p38γ$^{-/-}$ or p38δ$^{-/-}$ preadipocytes. A. Effect of U0126 (10µM) (n=4) or PD184352 (2µM) (n=5) pretreatment on IL6 expression in Wt preadipocytes. Western-blots and quantification of relative phosphorylation state of ERK1/2 (pERK1/2) in WT and p38γ$^{-/-}$ or p38δ$^{-/-}$ preadipocytes treated with U0126 (10 µM) (n=2) (B) or PD184352 (2µM) (n=4) (C). Total ERK1/2 and α-tubulin or Vinculin (loading control in each case) were used to the quantification of pERK1/2. D. Effect of U0126 (10 µM) (n=2) or PD184352 (2µM) (n=4) pretreatment on Wnt5a expression in p38γ$^{-/-}$ or p38δ$^{-/-}$ preadipocytes. P-value were obtained using unpaired two-tailed t-test or one-way ANOVA coupled to Bonferroni's post-test **p < 0.05, **p < 0.01, ***p < 0.001, ****p < 0.0001*.

Supplementary figure 6

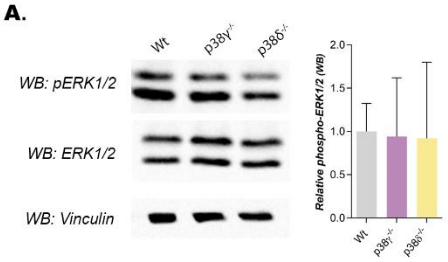

Supplementary Figure 6. Phosphorylation state of ERK1/2 in WT and p38$\gamma^{-/-}$ or p38$\delta^{-/-}$ preadipocytes. A. Western blot and quantification of phospho-ERK1/2 (pERK1/2) (n=4). Total ERK1/2 and Vinculin (loading control) were used to the quantification of pERK1/2. P-value were obtained using one-way ANOVA coupled to Bonferroni's post-test.

Supplementary figure 7

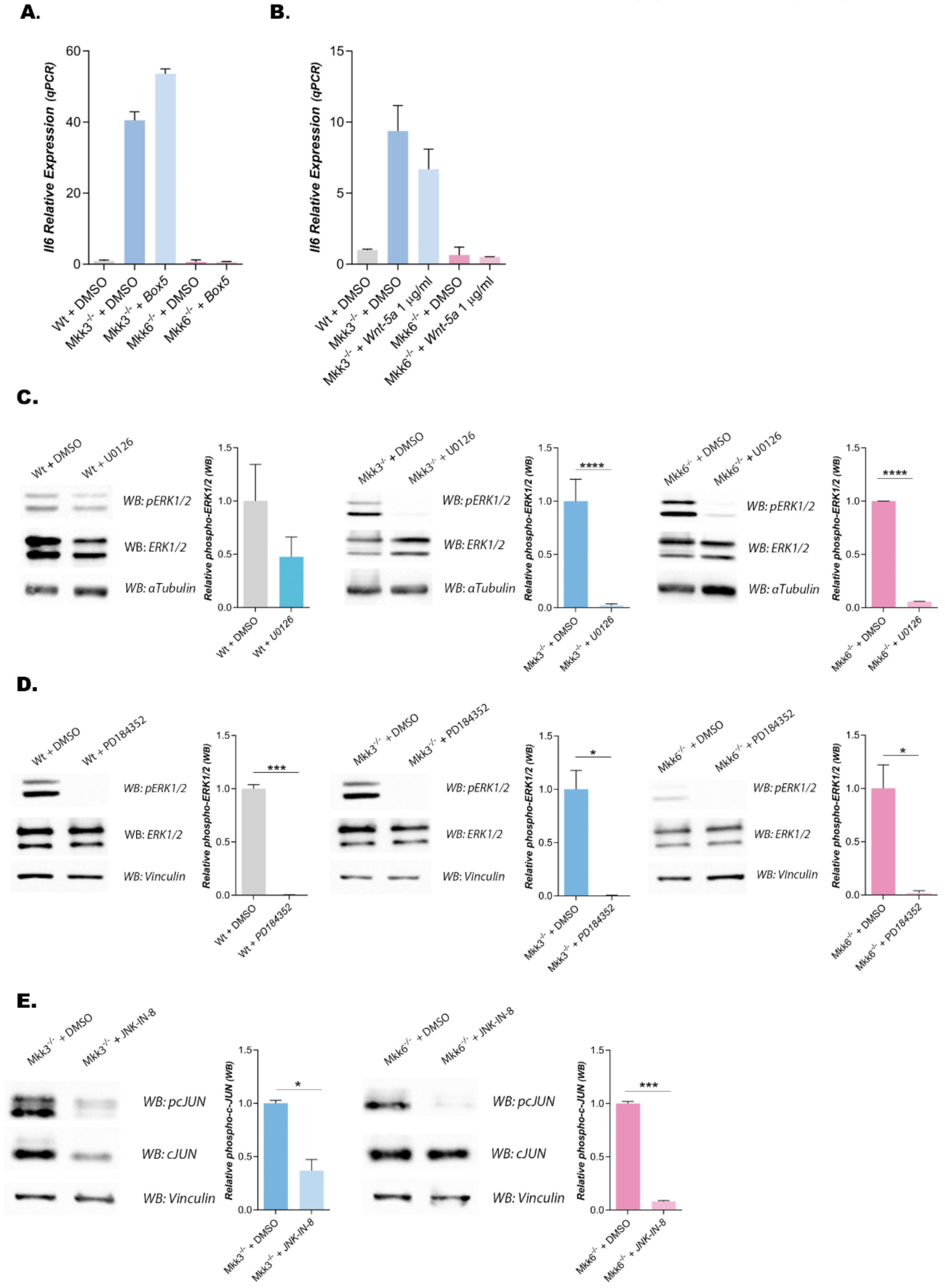

Supplementary Figure 7. IL6 expression and phosphorylation state of ERK1/2 and cJUN in WT, preadipocytes. (A) Effect of Box5 (3µg/ml) and exogenous Wnt5a (1µg/ml) (B) on IL6 expression (n=2). Western-blots and quantification of relative phosphorylation state of ERK1/2 (pERK1/2) in WT and Mkk3$^{-/-}$ or MKK6$^{-/-}$ preadipocytes treated with U0126 (10 µM) (n=2) (C) or PD184352 (2µM) (n=4) (D). Total ERK1/2 and α-tubulin or Vinculin (loading control in each case) were used to the quantification of pERK1/2. E. Western-blots and quantification of phospho-cJUN (pcJUN) in control MKK3$^{-/-}$ or MKK6$^{-/-}$ preadipocytes and treated with JNK-IN-8 (3µM) (n=4). Total cJUN and Vinculin (loading control) were used to the quantification of pcJUN. P-value were obtained using unpaired two-tailed t-test or one-way ANOVA coupled to Bonferroni's post-test. *$p < 0.05$, ***$p < 0.001$, ****$p < 0.0001$.*

Supplementary Table 1: SwissTargetPrediction BIRB0796 vs Mus Musculus. Probability of interaction between the ligand BIRB0796 and proteins obtained in Protein Target Prediction. Data referring to p38alpha, p38beta and JNK2 are highlighted in red and data referring to ERK2 are highlighted in green. Note that p38gamma and p38delta are absent in the list obtained in the prediction.

Target	Common name	ID	Target Class	Probability
**MAP Kinase p38 alpha**	Mapk14	P47811 CHEMBL2336	Kinase	**0.806139984034**
Tyrosine-protein Kinase ABL (by homology)	Abl1	P00520 CHEMBL3099	Kinase	0.708600275424
Macrophage colony-stimulating factor 1 receptor (by homology)	Csf1r	P09581 CHEMBL5570	Kinase	0.699816427534
**MAP Kinase ERK2 (by homology)**	Mapk1	P63085 CHEMBL2207	Kinase	**0.699816427534**
Mast/stem cell growth factor receptor Kit (by homology)	Kit	P05532 CHEMBL2034798	Kinase	0.656056327825
Serine/threonine-protein kinase B-raf (by homology)	Braf	P28028 CHEMBL2331061	Kinase	0.656056327825
**Mitogen-activated protein Kinase 9 (by homology)**	Mapk9	Q9WTU6 CHEMBL2034797	Kinase	**0.656056327825**
Protein Kinase C delta (by homology)	Prkcd	P28867 CHEMBL2560	Kinase	0.647335168236
Vascular endotelial growth factor receptor 1 (by homology)	Flt1	P35969 CHEMBL3516	Kinase	0.647335168236
Epidermal growth factor receptor erbB1 (by homology)	Egfr	Q01279 CHEMBL3608	Kinase	0.647335168236
RAF proto-oncogene serine/threonine-protein Kinase (by homology)	Raf1	Q99N57 CHEMBL3804748	Kinase	0.647335168236
Proto-oncogene tyrosine-protein Kinase receptor Ret (by homology)	Ret	P35546 CHEMBL2034799	Kinase	0.647335168236

Target	Common name	ID	Target Class	Probability
Tyrosine-protein Kinase BLK (by homology)	Blk	P16277 CHEMBL3343	Kinase	0.647335168236
Tyrosine-protein Kinase LCK (by homology)	Lck	P06240 CHEMBL2480	Kinase	0.647335168236
Tyrosine-protein Kinase Lyn (by homology)	Lyn	P25911 CHEMBL2258	Kinase	0.647335168236
**MAP Kinase p38 beta (by homology)**	Mapk11	Q9WUI1 CHEMBL4335	Kinase	**0.647335168236**
Fibroblast growth factor receptor 4 (by homology)	Fgfr4	Q03142 CHEMBL3839	Kinase	0.647335168236
STE20-like serine/ threonine-protein kinase(by homology)	Slk	O54988 CHEMBL2176844	Kinase	0.647335168236
Tyrosine-protein Kinase Fgr (by homology	Fgr	P14234 CHEMBL2034795	Kinase	0.647335168236
Tyrosine-protein kinase receptor Tie-1 (by homology)	Tie1	Q06806 CHEMBL2034800	Kinase	0.647335168236
Protein-tyrosine kinase 2-beta	Ptk2b	Q9QVP9 CHEMBL1075289	Kinase	0.0956237870388
